# Effects of *Chlorella* and *Spirulina* on bacterial community composition in a dual-flow continuous culture system

**DOI:** 10.1093/tas/txaf090

**Published:** 2025-07-11

**Authors:** E Sarmikasoglou, R R Lobo, L F Roesch, J R Vinyard, Z Yuting, K C C Jeong, C J Coronella, S R Hiibel, A P Faciola

**Affiliations:** Department of Animal Science, Michigan State University, East Lansing, MI, 48824, USA; Department of Animal Sciences, University of Florida, Gainesville, FL, 32611, USA; Department of Microbiology and Cell Science, University of Florida, Gainesville, FL, 32603, USA; Matanuska Experiment Farm and Extension Center, University of Alaska Fairbanks, Palmer, AK, 99645, USA; Department of Animal Sciences, University of Florida, Gainesville, FL, 32611, USA; Department of Animal Sciences, University of Florida, Gainesville, FL, 32611, USA; Department of Chemical and Materials Engineering, University of Nevada, Reno, NV 89557, USA; Department of Chemical and Materials Engineering, University of Nevada, Reno, NV 89557, USA; Department of Animal Sciences, University of Florida, Gainesville, FL, 32611, USA

**Keywords:** algae, *Chlorella*, rumen bacteria, *Spirulina*

## Abstract

The objective of this study was to evaluate the partial replacement of soybean meal (SBM) with either *Chlorella pyrenoidosa* or *Spirulina platensis* in a high producing dairy cow diet on ruminal bacterial communities. A dual-flow continuous culture system was used in a replicated 3 × 3 Latin Square design. A control diet (CRT) with SBM at 17.8% DM; and 50% SBM biomass replacement with either *Chlorella pyrenoidosa* (CHL); or *Spirulina platensis* (SPI). All diets were formulated to provide 16.0% CP, 34.9% NDF, 31.0% starch DM basis. Samples were collected from the fluid and solid effluents at 3, 6, and 9 h after feeding; a composite of all time points was made for each fermenter within their respective fractions. Treatment responses for bacterial community structure were analyzed with the PERMANOVA test run with the R *Vegan* package. Orthogonal contrasts were used to test the effects of 1) partial replacement of SBM with algae (CRT vs. CHL, and SPI); and 2) the comparison of algae sources (CHL vs. SPI). The orthogonal contrasts were used to test the effects of the treatments on phylum, family, and genus differential abundance using the R *limma* package. The relative abundance of *Ruminobacter* in liquid fraction was greater for CHL and SPI than CRT, while the relative abundance of *Butyrivibrio*, and *Pseudobutyrivibrio* in solid fraction were lower for CHL and SPI compared to CRT, respectively. Moreover, the relative abundance of *Ruminobacter* in liquid fraction was greater for CHL compared to SPI. Our results demonstrate that *Chlorella* and *Spirulina* supplementation enhance the abundance of bacteria associated with propionate production in the rumen.

## Teaser

*Chlorella* and *Spirulina* supplementation increased succinate producing when supplemented in a mid-lactation diet.

## INTRODUCTION

Soybean meal (**SBM**) constitutes the primary dietary protein source of dairy cattle diets in the United States due to its high protein content and good amino acid profile (NASEM, 2021). In 2022, approximately 4.2 million tons of SBM, equivalent to 11% of total SBM consumption by animal agriculture, were used by the dairy industry (United Soybean Board, 2023 ). However, dependency on a single feed ingredient, would become problematic due to potential trade distortions, availability, price variability (Zhang and Liu, 2020) as well as, sustainability issues related to monoculture (Jia et al., 2020). Moreover, raw soybeans contain anti-nutritional factors such as saponins, trypsin inhibitors, and phytates that are typically found in legume seeds. Therefore, alternative protein sources are of importance. *Spirulina* and *Chlorella* are microalgae genera with 50% to 70% protein (DM basis) and an amino acid profile comparable to other food proteins such as eggs and soybeans (Becker, 2007). Partial replacement of SBM with either *Chlorella pyrenoidosa* or *Spirulina platensis* exhibited no effects in rumen fermentation in a serum vial culture (Lobo et al., 2024a), and a reduction in branched short-chain fatty acids and isoacids that are biomarkers for ruminal protein degradation and potential improvement in nitrogen utilization in a continuous culture (Lobo et al., 2024b). Furthermore, these algae sources contain polyphenols, carotenoids, and polysaccharides with anti-inflammatory and anti-microbial activity against pathogenic bacteria (Zhang et al., 2018; Bortolini et al., 2022). *Spirulina* has a greater antioxidant capacity compared to *Chlorella* due to its higher content of polyphenols (Panaite et al., 2023). To better understand the feeding value of algal biomass in dairy cattle diets it is important to evaluate its effects on ruminal microbiome. Therefore, the objective of this study was to evaluate the partial replacement of SBM with two representative microalgae species, *Chlorella pyrenoidosa* or *Spirulina platensis*, on ruminal bacterial communities in a dual-flow continuous culture system using a high producing dairy cow diet.

## MATERIALS AND METHODS

### Ethical Approval

The University of Florida Institutional Animal Use and Care Committee approved all the animal care and handling procedures required for this experiment.

### Experimental Design, Diets, and Treatments

A detailed description of the design, diets, and treatments can be found in our companion study (Lobo et al., 2024b). Briefly, the design consisted of three fermentation periods of 10-d each consisting of 7-d of adaptation and 3-d of sampling. Six dual-flow continuous culture fermenters were used in a replicated 3 × 3 Latin square design. All diets were formulated according to the NASEM (2021) recommendations for a lactating Holstein cow with 680 kg body weight and milk production of 45 kg/d with 3.5% fat, 3.0% protein, and 4.8% lactose. Experimental diets where control (**CRT**) with SBM as the main protein source (17.8% of the diet DM), 50% replacement of SBM with *Chlorella pyrenoidosa* (**CHL**), and 50% replacement of SBM with *Spirulina platensis* (**SPI**). The inclusion level of either algae source was based on a previous dose response study where the replacement of SBM with CHL or SPI did not negatively affect ruminal fermentation (Lobo et al., 2024a). Both algae sources were delipidated with cracked cell walls and were purchased from Prime Chlorella Distribution Inc. (Calgary, AB, Canada). All feed ingredients were ground through a 2 mm screen in a Wiley mill (model N°2; Arthur H. Thomas Co., Philadelphia, PA). The corn silage was dried for 72 h at 60 °C in a forced-air oven (Heratherm, Thermo Scientific, Waltham, MA) to allow for partial dryness of the material before grinding. Subsamples from each individual ingredient were ground through a 2 mm sieve and sent to Dairy One Laboratory (Ithaca, NY) for chemical composition analyses. Each fermenter was provided its respective experimental diet (106 g/d DM) divided equally between two feedings at 0800 h and 1800 h. The CHL and SPI were added as dry products to their respective diets and divided into two equal doses respectively.

### Dual-Flow Continuous Culture System Operation

A dual-flow continuous culture system, as described by Hoover et al. (1976) and modified by Paula et al. (2017) was used for this experiment. Ruminal fermentation is simulated in this system through continuous agitation (100 rpm), infusion of N_2_ gas to displace oxygen, constant temperature (39 °C), and infusion of artificial saliva (Weller and Pilgrim, 1974) at 3.05 mL per min to individually regulate passage rates of liquid (11% h^−1^) and solid (5.5% h^−1^) effluents of digesta.

This experiment consisted of 3 fermentation periods of 10-d each (30-d of in vitro fermentation total). On d-1 of each fermentation period the fermenters were inoculated with ruminal contents collected from 3 ruminally cannulated Holstein cows in mid lactation fed twice daily a TMR with 60% corn silage, 12.5% ground corn, 13% citrus pulp, 12% SBM, and 2.5% mineral and vitamin premix (DM basis). Ruminal digesta was collected from the ventral, central, and dorsal areas of the rumen, strained through 4 layers of cheesecloth and transferred into pre-warmed thermoses. Ruminal content from all cows was homogenized and 1.82 L was inoculated to each fermenter. Fermenters were pre-warmed and under continuous flush of N_2_ gas during inoculation.

### Experimental Procedure and Sampling

Each experimental period consisted of three 10-d of in vitro fermentation. The first 7-d of fermentation of each period were used for adaptation to experimental diets and stabilization of bacterial communities (Salfer et al., 2018). Samples for bacterial sequencing analysis were collected on d-8,9,10 from both liquid and solid effluents of each fermenter at 3, 6, and 9 h post morning feeding daily. For the liquid fraction, 5 mL of liquid effluent were collected at each timepoint, totaling 45 mL per fermenter per period, resulting in 6 samples per period. For the solid fraction, 22 g of solid effluent were collected at each timepoint and strained through four layers of cheesecloth, totaling approximately 200 g of solid sample collected from each fermenter per period, resulting in 6 samples per period. Upon collection, samples were stored at −80 °C for subsequent DNA extraction.

### DNA Extraction, Polymerase Chain Reaction Amplification, and rRNA Sequencing

Total genomic DNA from ruminal samples were extracted using the Quick-DNA Fecal/Soil Microbe Miniprep Kit (D6010, Zymo Research Corporation, Irvine, CA, USA), following the manufacturer’s instructions. Before storage in −80 °C, the extracted DNA concentration was measured using a Qubit Fluorometer (Invitrogen Waltham, MA, USA). DNA sequencing procedures were performed according to Kozich et al. (2013). Amplification with PCR was performed in a C1000 Touch Thermal Cycler (Bio-Rad Laboratories, Hercules, CA, USA). The V4 region of the 16S rRNA gene was amplified by dual-index universal bacterial primers (515F: 5′-GTGCCAGCMGCCGCGGTAA-3′; 806R: 5′-GGACTACHVGGGTWTCTAAT-3′; Caporaso et al., 2011) through an initial denaturation of 5 min under 95 °C, followed by 30 cycles of 30 s at 95 °C, 30 s at 55 °C, 1 min at 72 °C, and 5 min for final elongation at 72 °C. Forward and reverse primers, as well as small DNA fragment contaminants, were removed using a 1% low-melting agarose gel extraction kit. Amplicons were then purified and normalized using a SequalPrep plate kit (Invitrogen Waltham, MA, USA), and the DNA concentration was measured with a Qubit fluorometer (Invitrogen Waltham, MA, USA). Adapters were added to the amplicons, and the DNA library was constructed by equally pooling all the amplicons together and using quantitative real-time PCR for quality check. Sequencing was performed using a MiSeq reagent kit V2 (2 × 250 cycles run; Illumina) in an Illumina MiSeq platform at the Interdisciplinary Center for Biotechnology Research at the University of Florida (Gainesville, FL, USA). Sequences were deposited at the Sequence Read Archive of the National Center for Biotechnology Information (https://www.ncbi.nlm.nih.gov/sra) under access no. *PRJNA1169366.*

### Bioinformatics and Analyses

Sequenced amplicons were processed using the DADA2 pipeline (version 1.16) in R (Callahan et al., 2016), and the taxonomy assignment was performed using the Bayesian RDP classifier trained with the RDP train set 18 database (Cole et al., 2014; Edgar, 2018). Briefly, paired-end raw reads were demultiplexed, and the quality profiles of the forward and reverse readings were separately inspected, filtered, and trimmed based on the relationship between error rates and quality scores. Amplicons were truncated at 30 bp to remove the forward primer. Reads with at least one ambiguous nucleotide were filtered out. The maximum error allowed in a read was set to 2. Forward and reverse readings were merged, chimeras removed, and an amplicon sequence variants table was created. The resulting tables were converted into a phyloseq object for downstream analyses. (McMurdie and Holmes, 2013). Before further data analysis, we calculated the coverage of the dataset according to Good (1953) to evaluate whether the number of sequences obtained for each sample was adequate to provide representativeness of the bacterial community (Supplemental Table S1; 10.6084/m9.figshare.27270207). To protect against ASVs with small mean and large coefficient of variation, we first removed ASVs not seen more than 3 times in at least 20% of the samples and then rarefied the dataset. After rarefying the dataset at 1,984 sequences (equivalent to the sample with the smallest number of sequences), all samples had coverage > 99% and thus were considered representative. Sequencing depth was normalized by the minimum library size (1,984 sequences per sample) to perform all microbiome analyses.

### Statistical Analyses

Bacterial alpha diversity indices (Chao1 and Shannon) were calculated with R *phyloseq* package (McMurdie and Holmes, 2013). Bacterial community structure, using the Bray-Curtis dissimilarity, was visualized by Principal Coordinates analysis (**PCoA**), and the statistical differences among samples were measured by PERMANOVA using the *vegan* R package (Oksanen, 2007). Orthogonal contrasts were used to test the effects of 1) partial replacement of SBM with the microalgae sources (CRT vs. CHL, and SPI); and 2) the comparison of algae sources (CHL vs. SPI). The orthogonal contrasts were used to test the effects of the treatments on phylum, family, and genus differential abundance using the R *limma* package (Ritchie et al., 2015). Significance was declared at *P* ≤ 0.05.

## RESULTS AND DISCUSSION

### Effects on Rumen Bacterial Community

**Figure 1.**
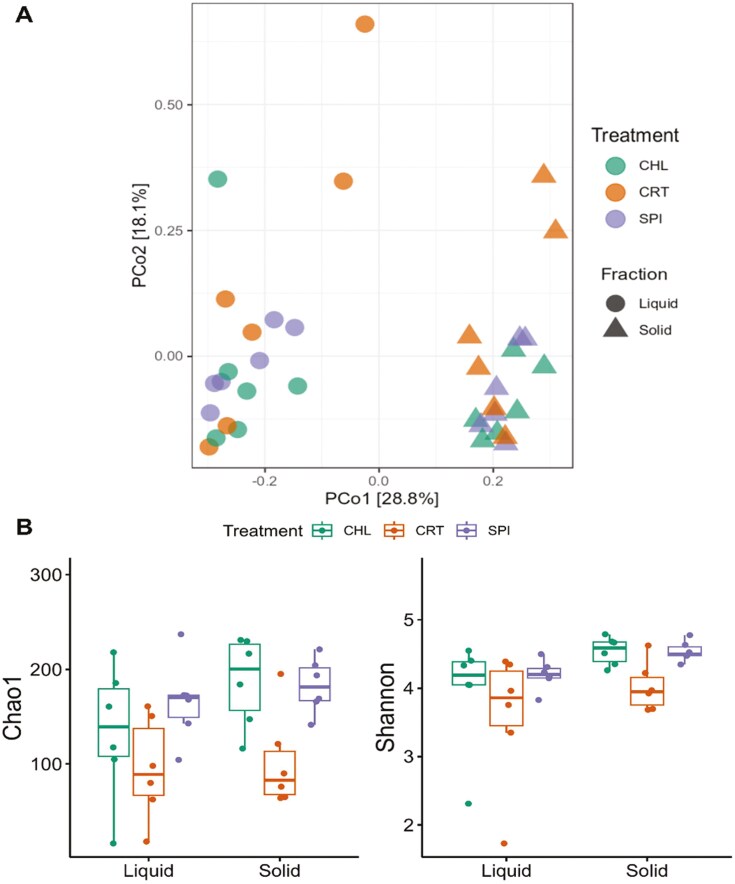
Principal coordinates analysis (PCoA) plots of Bray-Curtis dissimilarity matrix at ASV level comparing the treatment effects on community structure of ruminal bacteria (**A**), effects of *Chlorella* and *Spirulina* in richness and diversity of the ruminal bacterial community in dual-flow continuous culture (**B**). The PERMANOVA analysis indicated that the fraction type (liquid or solid) contributed to 27.3% of the variation in community distances (*P* < 0.01). Effects of *Chlorella* and *Spirulina* in richness and diversity of the ruminal bacterial community in dual-flow continuous culture. Experimental diets where control (**CRT**) with soybean meal as the main protein source, 50% replacement of soybean meal with *Chlorella pyrenoidosa* (**CHL**), and 50% replacement of soybean meal with *Spirulina platensis* (**SPI**). Contrasts were used to test the effects of 1) partial replacement of soybean meal with algae (CRT vs. CHL, and SPI); and 2) the comparison of algae sources (CHL vs. SPI). Error bars indicate the SE.

The microbial community structure is reported in Figure 1A. Principal coordinates analysis shows clustering in the microbiome of the liquid and solid fractions of fermentation but, no difference related to the treatments were observed regarding the community structure suggesting that the treatments did not affect the bacterial beta diversity. Moreover, the PERMANOVA analysis indicated that the fraction type (liquid or solid) contributed to 27.3% of the variation in community distances (*P* < 0.01). Previous studies, consistent with our findings, have reported the presence of bacteria in the free floating fraction co-habiting with others attached to particles (Czerkawski, 1986; Henderson et al., 2013). The richness and diversity at the ASV level of the bacterial community are presented in Figure 1B. Regarding the bacterial richness (Chao1), and diversity (Shannon), no effects were detected in any of the orthogonal contrasts tested for either liquid or solid fraction, respectively. The absence of effects suggests that the microbial diversity remains unaffected by the inclusion of either algal source. Previously, spirulina supplementation has been reported to not affect the ruminal bacterial richness and diversity at inclusion rate of 3% DM in Hu sheep fed a high fat diet (Wang et al., 2023). Further we analyzed the effects from algal sources on ruminal microbiome, at phylum, family, and genus levels.

### Effects on Bacterial Community Phyla, Families, and Genera

A total of 6 phyla, 11 families, and 13 genera were identified in both the liquid (Table 1) and solid (Table 2) fractions that exhibited a relative abundance greater than 3%. The main phyla in both fractions were *Firmicutes*, and *Bacteroidetes*, which is in accordance with previous studies evaluating the rumen core microbiome in cattle under predominantly forage-based diets (Henderson et al., 2015; McCann et al., 2016). Among the reported phyla no treatment effects were detected in both fractions (all *P* > 0.05). In family level, the relative abundance of *Succinivibrionaceae* (*P* = 0.02) in liquid fraction (Table 1), and *Ruminococcaceae* (*P* = 0.01) in solid fraction (Table 2) were greater for CHL and SPI compared to CRT, while the relative abundance of *Selenomonadaceae* (*P* = 0.01) in solid fraction (Table 2) was lower for CHL and SPI compared to CRT. In genus level, the relative abundance of *Ruminobacter* (*P* < 0.01) in liquid fraction (Table 1) was greater for CHL and SPI compared to CRT, while the relative abundance of *Butyrivibrio* (*P* < 0.01), and *Pseudobutyrivibrio* (*P* = 0.01) in solid fraction (Table 2) were lower for CHL and SPI compared to CRT, respectively. Furthermore, the relative abundance of *Ruminobacter* (*P* = 0.03) in liquid fraction was greater for CHL compared to SPI (Table 1).

**Table 1.** Effects of *Chlorella* and *Spirulina* on relative abundance of ruminal bacterial community composition in liquid fraction at phylum, family, and genus level

	Diet^1^				Contrasts *P*-value^2^	
Items	CRT	CHL	SPI	SEM	Control vs. algae	CHL vs. SPI
Phylum						
*Firmicutes*	34.1	28.9	30.9	2.76	0.41	0.57
*Bacteroidetes*	25.5	28.1	30.7	3.75	0.40	0.47
*Proteobacteria*	24.6	26.2	21.9	3.83	0.60	0.49
*Spirochaetes*	7.92	8.93	10.2	1.19	0.37	0.47
Family						
*Prevotellaceae*	19.7	20.3	25.5	2.72	0.74	0.20
*Spirochaetaceae*	10.9	12.2	13.7	1.59	0.57	0.59
*Clostridiaceae_1*	13.2	8.36	9.87	3.22	0.86	0.36
*Lachnospiraceae*	9.48	7.23	11.2	1.61	0.88	0.14
*Succinivibrionaceae*	5.28	14.8	7.34	1.66	0.02	0.10
*Selenomonadaceae*	13.7	5.84	7.17	2.57	0.51	0.76
*Ruminococcaceae*	6.68	8.28	7.09	1.67	0.90	0.34
*Acidaminococcaceae*	3.17	6.8	3.26	1.67	0.76	0.61
*Methanobacteriaceae*	4.94	3.26	3.17	0.66	0.54	0.98
Genus						
*Prevotella*	22.4	22.1	27.2	2.94	0.84	0.24
*Clostridium_sensu_stricto*	15.3	9.70	12.3	3.67	0.85	0.36
*Treponema*	10.9	12.7	13.6	1.70	0.39	0.63
*Selenomonas*	11.0	4.99	6.71	1.97	0.52	0.70
*Succinivibrio*	5.01	7.60	6.13	1.12	0.58	0.46
*Succiniclasticum*	3.07	7.61	3.95	1.80	0.29	0.60
*Ruminobacter*	1.68	9.96	2.92	1.85	<0.01	0.03
*Methanobrevibacter*	5.66	3.88	3.80	0.73	0.50	0.99
*Fibrobacter*	3.79	3.73	3.02	1.06	0.77	0.62
*Clostridium_*IV	2.98	3.97	2.38	1.20	0.53	0.78

^1^Experimental diets where control (**CRT**) with soybean meal as the main protein source, 50% replacement of soybean meal with *Chlorella pyrenoidosa* (**CHL**), and 50% replacement of soybean meal with *Spirulina platensis* (**SPI**).

^2^Contrasts were used to test the effects of 1) partial replacement of soybean meal with microalgae (CRT vs. CHL, and SPI); and 2) the comparison of algae sources (CHL vs. SPI).

**Table 2.** Effects of *Chlorella* and *Spirulina* on relative abundance of ruminal bacterial community composition in solid fraction at phylum, family, and genus level

	Diet^1^				Contrasts *P*-value^2^	
Items	CRT	CHL	SPI	SEM	Control vs. algae	CHL vs. SPI
Phylum						
*Firmicutes*	40.5	36.6	37.6	2.12	0.20	0.75
*Bacteroidetes*	18.0	20.8	20.8	1.13	0.20	0.89
*Spirochaetes*	18.9	21.4	20.2	2.43	0.40	0.78
*Proteobacteria*	8.40	8.79	9.05	1.02	0.58	0.94
*Euryarchaeota*	8.53	5.88	6.09	1.40	0.29	0.88
*Fibrobacteres*	4.52	4.48	4.88	0.67	0.53	0.80
Family						
*Lachnospiraceae*	26.1	22.8	23.1	1.36	0.09	0.93
*Spirochaetaceae*	21.2	24.3	22.8	2.86	0.38	0.75
*Prevotellaceae*	12.1	14.9	15.3	1.32	0.13	0.95
*Methanobacteriaceae*	9.44	6.73	6.84	1.59	0.30	0.94
*Selenomonadaceae*	8.44	4.46	5.04	0.97	0.01	0.74
*Fibrobacteraceae*	5.20	5.29	5.70	0.79	0.40	0.83
*Ruminococcaceae*	2.95	5.52	4.69	0.55	0.01	0.61
*Acidaminococcaceae*	2.89	4.94	4.98	1.07	0.25	0.80
*Bacteroidaceae*	4.20	3.36	2.89	0.48	0.14	0.49
Genus						
*Treponema*	22.0	26.6	24.6	2.79	0.16	0.69
*Prevotella*	13.5	17.1	17.4	1.36	0.06	0.90
*Methanobrevibacter*	9.94	7.28	7.46	1.70	0.37	0.89
*Fibrobacter*	6.27	6.61	7.05	1.01	0.35	0.89
*Lachnobacterium*	7.59	5.66	6.36	1.01	0.28	0.66
*Butyrivibrio*	7.93	5.57	4.68	0.45	<0.01	0.36
*Succiniclasticum*	2.97	5.95	5.93	1.27	0.09	0.83
*Pseudobutyrivibrio*	5.07	3.67	2.88	0.45	0.01	0.32

^1^Experimental diets where control (**CRT**) with soybean meal as the main protein source, 50% replacement of soybean meal with *Chlorella pyrenoidosa* (**CHL**), and 50% replacement of soybean meal with *Spirulina platensis* (**SPI**).

^2^Contrasts were used to test the effects of 1) partial replacement of soybean meal with microalgae (CRT vs. CHL, and SPI); and 2) the comparison of algae sources (CHL vs. SPI).

Both algae sources increased the abundance of succinate producers including genera of *Succinivibrionaceae* family, such as *Ruminobacter*, while it reduced the abundance of succinate utilizers from *Selenomonadaceae* family. Similar to our findings, previous studies that supplemented 0.2 g *Asparagopsis taxiformis* in vitro by using the rumen simulation technique (RUSITEC) exhibited an increase in *Ruminobacter*, and a decrease in *Selenomonas* abundance (O’Hara et al., 2023). Succinate is the major precursor of the propionate synthesis in the rumen, and it seems that algae supplementation affects succinate utilizers from *Selenomonadaceae* family differently. Based on the increase in propionate production that we observed in our companion paper (Lobo et al., 2024b), we would suggest that some genera from *Selenomonadaceae* family are suppressed while others, such as *Succiniclasticum* (*P* = 0.09) enhanced. These variations would potentially be attributed to selective inhibitory activity of polyphenols, carotenoids, and/or polysaccharides included with our microalgae sources. However, further research needs to elucidate algae effect on species level. Regarding lignocellulose fermentation, the algae supplementation it seems to be family specific. More specifically, it promotes members of *Ruminococcaceae*, while it inhibits species of *Lachnospiraceae*, such as *Butyrivibrio*, and *Pseudobutyrivibrio. Butyrivibrio*, and *Pseudobutyrivibrio* that are known to ferment carbohydrates to butyrate, formate, lactate, and acetate (Kopečný et al., 2003; Moon et al., 2008), which explains the decrease in butyrate production from our companion study (Lobo et al., 2024b). The growth of lactic acid bacteria isolated from sheep feces was inhibited by plant extracts rich in phenolic compounds (Salem et al., 2010). Similarly, the flavonoids and phenolic acids present in our microalgae sources may inhibit carbohydrate-fermenting species from the *Lachnospiraceae* family that produce organic acids.

## CONCLUSIONS

In conclusion, partial replacement of SBM with *Chlorella* or *Spirulina* increased the relative abundance of *Ruminobacter*, and decreased the relative abundance of *Butyrivibrio*, and *Pseudobutyrivibrio*. Moreover, *Chlorella* enhanced the relative abundance of *Ruminobacter* compared to *Spirulina*. Overall, the algae sources tested seem to affect the bacteria related to the propionate production and fiber fermentation in the rumen.

## Supplementary Material

txaf090_suppl_Supplementary_Tables_S1
