# Supplementary material for: Effects of Chlorella and Spirulina on bacterial community composition in a dual-flow continuous culture system
Source: Transl Anim Sci. 2025 Jul 11;9:txaf090. doi: 10.1093/tas/txaf090 (PMC12272054; doi:10.1093/tas/txaf090)
Supplement: txaf090_suppl_Supplementary_Tables_S1 [file txaf090_suppl_supplementary_tables_s1.docx]

**Effects of *Chlorella* and *Spirulina* on bacterial community composition in a dual-flow continuous culture system**

**E. Sarmikasoglou,^1^ R. R. Lobo,^2^ L. F. Roesch,^3^ J. R. Vinyard,^4^ C. J. Coronella,^5^ S. R. Hiibel,^5^ and A. P. Faciola^2^***

^1^Department of Animal Science, Michigan State University, East Lansing, MI, 48824, USA

^2^Department of Animal Sciences, University of Florida, Gainesville, FL, 32611, USA

^3^Department of Microbiology and Cell Science, University of Florida, Gainesville, FL, 32603, USA

^4^Matanuska Experiment Farm and Extension Center, University of Alaska Fairbanks, Palmer, AK, 99645, USA

^5^Department of Chemical and Materials Engineering, University of Nevada, Reno, NV 89557, USA

^2^Corresponding author: [afaciola@ufl.edu](mailto:afaciola@ufl.edu)

**Supplemental Table 1.** Distribution of reads per sample.

| Sample | Treatment | Total Reads | Rarefied Reads | Coverage after rarefaction |
| --- | --- | --- | --- | --- |
| R1_S248_L001 | CRT | 4062 | 1984 | 1 |
| R10_S257_L001 | SPI | 11404 | 1984 | 0.992943548 |
| R12_S259_L001 | CRT | 10034 | 1984 | 0.993951613 |
| R13_S260_L001 | CRT | 6118 | 1984 | 0.998991935 |
| R15_S2_L001 | SPI | 8578 | 1984 | 0.993447581 |
| R16_S3_L001 | CHL | 5731 | 1984 | 0.994455645 |
| R17_S4_L001 | SPI | 7170 | 1984 | 0.990927419 |
| R18_S5_L001 | CRT | 3652 | 1984 | 1 |
| R19_S6_L001 | CHL | 7107 | 1984 | 0.996975806 |
| R2_S249_L001 | CHL | 1984 | 1984 | 1 |
| R22_S9_L001 | SPI | 8670 | 1984 | 0.989919355 |
| R23_S10_L001 | CHL | 6686 | 1984 | 0.997983871 |
| R24_S11_L001 | CRT | 5350 | 1984 | 0.997479839 |
| R25_S12_L001 | CRT | 4995 | 1984 | 1 |
| R26_S13_L001 | CHL | 10828 | 1984 | 0.987399194 |
| R27_S14_L001 | SPI | 9471 | 1984 | 0.993951613 |
| R3_S250_L001 | SPI | 10316 | 1984 | 0.982862903 |
| R30_S17_L001 | CHL | 8748 | 1984 | 0.989415323 |
| R31_S18_L001 | CRT | 7829 | 1984 | 0.998487903 |
| R32_S19_L001 | SPI | 9660 | 1984 | 0.987399194 |
| R33_S20_L001 | CHL | 9706 | 1984 | 0.985887097 |
| R34_S21_L001 | SPI | 10085 | 1984 | 0.986391129 |
| R36_S23_L001 | CRT | 6975 | 1984 | 0.999495968 |
| R37_S24_L001 | CRT | 5494 | 1984 | 1 |
| R39_S26_L001 | SPI | 10905 | 1984 | 0.992943548 |
| R40_S27_L001 | CHL | 11419 | 1984 | 0.986391129 |
| R41_S28_L001 | SPI | 10199 | 1984 | 0.98891129 |
| R42_S29_L001 | CRT | 10872 | 1984 | 0.990423387 |
| R43_S30_L001 | CHL | 7283 | 1984 | 0.998487903 |
| R46_S33_L001 | SPI | 7036 | 1984 | 0.996471774 |
| R47_S34_L001 | CHL | 7703 | 1984 | 0.993951613 |
| R48_S35_L001 | CRT | 6285 | 1984 | 0.997479839 |
| R6_S253_L001 | CHL | 10120 | 1984 | 0.991431452 |
| R7_S254_L001 | CRT | 9827 | 1984 | 0.994959677 |
| R8_S255_L001 | SPI | 7697 | 1984 | 0.998487903 |
| R9_S256_L001 | CHL | 11310 | 1984 | 0.985887097 |
